# Multidimensional complexities of filariasis control in an era of large-scale mass drug administration programmes: a can of worms

**DOI:** 10.1186/1756-3305-7-363

**Published:** 2014-08-15

**Authors:** David H Molyneux, Adrian Hopkins, Mark H Bradley, Louise A Kelly-Hope

**Affiliations:** Centre for Neglected Tropical Diseases, Department of Parasitology, Liverpool School of Tropical Medicine, Pembroke Place, Liverpool, L3 5QA UK; Mectizan Donation Program, Task Force for Global Health, Decatur, Atlanta, GA USA; GlaxoSmithKline, Global Health Programme, Brentford, UK

**Keywords:** Onchocerciasis, Lymphatic filariasis, LF, *Loa loa*, Loiasis, Soil transmitted helminths, STHs, Malaria, Mass drug administration, Albendazole, Ivermectin, Bed nets

## Abstract

The impact of control and elimination programmes by mass drug administration (MDA) targeting onchocerciasis and lymphatic filariasis (LF) in sub-Saharan Africa over the last two decades has resulted in significantly reduced prevalence and intensity of infection, with some areas interrupting transmission. However, given that these infections are often co-endemic and the drugs (either ivermectin alone or combined with albendazole) also impact on soil transmitted helminths (STH), the importance of this, in terms of reaching the global goals has not been assessed. The additional problem posed by *Loa loa,* where ivermectin cannot be safely administered due to the risk of serious adverse events compounds this situation and has left populations drug naïve and an alternative strategy to eliminate LF is yet to be initiated at scale. Here, we present a series of operational research questions, which must be addressed if the effectiveness of integrated control of filarial and helminth infections is to be understood for the endgame. This is particularly important in the diverse and dynamic epidemiological landscape, which has emerged as a result of the long-term large-scale mass drug administration (or not). There is a need for a more holistic approach to address these questions. Different programmes should examine this increased complexity, given that MDA has multiple impacts, drugs are given over different periods, and programmes have different individual targets.

## Background to large-scale programmes

The filarial infections of humans in Africa demonstrate a wide spectrum of pathology and epidemiology as well as a diversity of vectors, ecology and distribution [1]. Imposed on this already complex pattern have been large-scale intervention programmes, ongoing for 40 years, initially for onchocerciasis, and since 2000 for lymphatic filariasis (LF) [2–7]. These programmes have reduced the public health importance of onchocerciasis and LF in many parts of Africa including interrupting transmission in some areas [4, 8–12] through mass drug administration (MDA) as well as vector control, or a combination of both approaches [13–16].

The Onchocerciasis Control Programme in West Africa (OCP) launched in 1974 [17] was initially considered to be addressing a relatively homogenous and well defined epidemiological situation. However, gradually during the period of its operations, research studies on blackfly (*Simulium ssp.*) populations demonstrated significant ecological and epidemiological complexity [18–20]. The drug Mectizan® (ivermectin-MSD) donated by Merck & Co Inc., was added as an annual treatment to the OCP interventions in 1988. This produced, where used, a rapid beneficial impact on morbidity and the progression of ocular lesions, thus preventing many cases of irreversible blindness as well as reducing the required duration of vector control by some two years [2]. Donated ivermectin was the cornerstone intervention used by the African Programme of Onchocerciasis Control (APOC) established in 1995 [21] (and by the Onchocerciasis Elimination Programme in the Americas (OEPA) [22–24]. At the time, APOC developed a sustainable delivery platform for ivermectin by adopting a policy of community directed delivery of ivermectin (CDTi) [25]. The objective was to facilitate the long term sustainable control of the public health problem due to onchocerciasis whilst reducing ocular and skin disease morbidity [5]; however, elimination was not considered to be a feasible goal at the time [26].

## Diversity of settings, epidemiology and interventions

The diversity of the settings in which the OCP was required to work in and deliver interventions [20], led to the concept of control epidemiology, which required a varied response to the endgame of OCP depending on the eco-geography of the vector and parasite (*Simulium spp. /Onchocerca volvulus)* complexes throughout the 11 countries of the OCP in West Africa. This was due to the different vectorial capacity of local *Simulium* species/cytoforms, defined by annual transmission potentials, initial prevalence levels and intensities as measured by community microfilarial loads and the varied pathology of forest and savannah forms of *O. volvulus*[2]. Over the last decade MDA programmes for onchocerciasis, LF and soil- transmitted helminths (STHs) have expanded in sympatric/co-endemic filarial infection settings throughout Africa [7, 27, 28]. This increased diversity of the epidemiology (ecology, zoogeography, vector characteristics, infection prevalence and intensity) needs to be addressed in a comprehensive way in the context of integrated neglected tropical disease (NTD) control/elimination.

Particular regard needs to be paid to coordinated mapping, and the dynamics created by ongoing interventions using drugs, which have an impact on filarial and helminth infections. This is important as individual disease specific programmes have expanded or are reaching endpoints, whilst there is the drive to integrate MDA using the preventive chemotherapy (PC) strategy for other infections such as trachoma and schistosomiasis [26]. For example, in Niger and some areas of Uganda where all indications are that onchocerciasis has been eliminated, treatment is ongoing with ivermectin and albendazole to complete the requirements of the LF programme. The reverse is true in the few countries where LF programmes have achieved the endpoints and treatment campaigns have stopped. Post-treatment surveillance (PST) will be a critical operation for national health systems to ensure that the public health gains, achieved at great cost over many years are not lost to undetected recrudescence.

**Figure 1:**
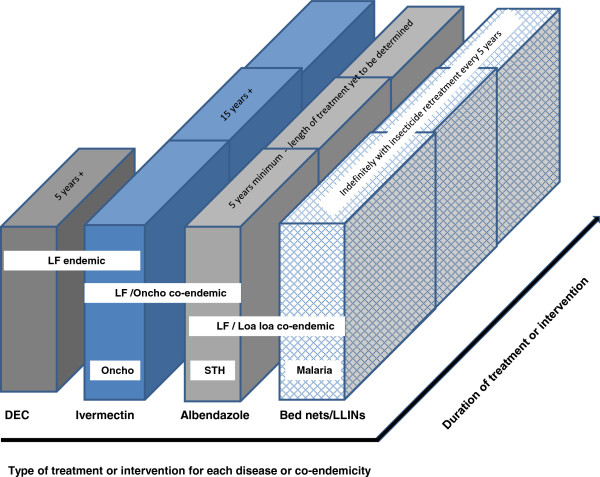
**Cross-cutting treatments and intervention for helminthiases (filarial and soil–transmitted) and malaria.** For STH moderate endemic areas (prevalence >20%) once yearly albendazole treatment is given, and in high endemic areas (prevalence >50%) twice yearly albendazole treatment is given. For LF*/L loa* co-endemic areas twice yearly albendazole treatment is given together with bed nets/LLINs.

The original epidemiological patterns of individual disease prevalence and intensity have thus become more diverse and complex as multiple control/elimination strategies, deploying a variety of drug combinations, with differential efficacies have been initiated and expanded over time. Levels of population coverage vary, as do target populations, and in the case of LF, the impact on transmission will vary if the programme overlaps with a previously treated onchocerciasis area or bed net/ long lasting insecticidal nets (LLINs) distribution for malaria control where over many areas the diseases are co-endemic [29]. In sub-Saharan Africa, initially control, and now the elimination of onchocerciasis and of LF has been targeted in parallel with the expansion of integrated MDA using the PC strategy, which also targets STH, schistosomiasis and trachoma [28]. MDA delivered through communities or via school based programmes targeting school age children are the core modes of delivery for STH and schistosomiasis. The drugs used for these programmes in sub-Saharan Africa are ivermectin, albendazole, mebendazole, praziquantel and azithromycin - all donated by pharmaceutical companies and are on the WHO Essential Medicine drugs list [3, 30]. Figure 1 highlights the complexity of cross-cutting treatment and interventions for helminthiases, which need to consider multiple epidemiological scenarios and include malaria vector control as either an alternative or additional strategy in certain co-endemic situations [13, 29].

The wider impacts of some of these drugs, the diversity of the disease ecology, extent of co-endemicity, the duration of targeted treatments required to eliminate some infections such as onchocerciasis and LF, as well as the complexity of the inter-disease epidemiology creates significant conceptual problems. To date the NTD community has tended to seek answers to unique questions, which relate to one or two diseases. This is best exemplified in the case of LF, where the impact of previous distributions of ivermectin, albendazole and bed nets/LLINs need to be investigated [13, 31, 32]. There has been limited recognition of the extent of the distribution of bed nets/LLINs over the last decade and the potential impact they may have had, in the absence of MDA in some LF endemic areas [13–16, 29]. A further consideration of the bed nets/LLINs on LF (as in malaria) will be the patterns of insecticide resistance, which will in the future add to the complexity of monitoring and evaluating LF programmes [33, 34]. This has not previously been considered.

There is also recognition that there is a need for an effective macrofilaricide to kill or permanently sterilise adult worms. The evidence that doxycycline is effective as it targets *Wolbachia* endosymbionts in both *Onchocerca* and *Wuchereria* has been known for over a decade [35]. However, there is now an opportunity to operationalise this approach albeit on a limited scale under medical supervision, whilst research to reduce the duration of antibiotic treatments and find additional suitable antibiotics continues. Studies in Cameroon have shown that there is high compliance by communities to a doxycycline regime despite a four week duration [36]. This should be exploited in areas/hotspots where there is suggested reduced efficacy of ivermectin or albendazole for both diseases, or predicted rates of decline of prevalence have not been in accord with those expected. There is also a need to introduce twice a year ivermectin, as has been the approach in the OEPA programme, in certain sub-Saharan Africa settings where this twice yearly regime may be better able to reduce the duration of annual treatments to achieve elimination of transmission of *O. volvulus*[22–24]. We summarise below the key operational research questions we consider must be addressed over the coming years in LF/onchocerciasis co-endemic areas.

The key questions that can be posed to address the multidimensional complexity of the current problems of MDA in LF/ onchocerciasis co-endemic areas (not *L. loa* endemic).

**Impact of MDAs on non-targeted infections**

- ➢*Some 20 project areas of APOC have reached a level where transmission (as measured by entomological studies and mf prevalence) has been reduced to a level compatible with achieving elimination of onchocerciasis; many of these areas are likely to have had endemic Wuchereria bancrofti; what has been the impact of ivermectin given annually for 15 years on endemic LF?*
- ➢*What has been the impact of ivermectin alone and with albendazole MDA for onchocerciasis and LF on STH and scabies*[12]*?; how has this impacted on mapping STH and on the prioritisation of interventions?*

**Onchocerciasis elimination and LF: the new questions**

- ➢*What is the geographic extent of the onchocerciasis low transmission zones and the population to be covered (hypoendemic areas previously untreated with ivermectin), which now need to be treated if onchocerciasis elimination is to be achieved, and how do they overlap with LF?*
- ➢*Have the hypoendemic onchocerciasis areas already been subjected to MDA for LF (where, when and for how long) and if so, what impact has that intervention had on onchocerciasis prevalence and intensity; has it been eliminated or prevalence reduced?*

**New strategies to address elimination: when, where, who and how**

- ➢*What is the feasibility of introducing twice yearly treatments of ivermectin to reduce the duration of treatment to achieve elimination of onchocerciasis faster (cf OEPA); what will be the impact on questions posed above; where should this approach be tested in Africa? What impact would this strategy have on LF?*
- ➢*Under what circumstances should doxycycline (or other anti Wolbachia antibiotics) be introduced as a macrofilaricidal strategy; what is needed to initiate such approaches, where and at what scale?*

**Impact of bed nets/LLINs**

- ➢*What has been the impact of bed nets/LLINs on their own or concurrently with ivermectin MDA on the distribution and prevalence of LF, and are LF specific interventions still needed*[13–16, 29]*?*
- ➢*What is the extent of the areas covered by bed nets /LLINs where LF had low endemicity/prevalence levels (implementation units prevalence circa 1-5%) as recorded during initial mapping studies*[37–39]*, and do these areas now need MDA for LF given the threshold level of 1% as the recommendation for an intervention for 5–7 years. If not, what is required to confirm elimination?*
- ➢*How long will LLINs remain efficacious as insecticide resistance to pyrethroids increases; what impact will a reduced LLIN efficacy have on the LF endgame*[33, 34]*?*

**STH interventions, strategies and interactions**

- ➢*Do the STH school based delivery strategies require modification given the impact of ivermectin and albendazole on STH where the drugs have been used for onchocerciasis and filariasis control?*
- ➢*To what extent, if any, has the treatment of adults within the onchocerciasis and LF programmes for extended periods impacted on transmission of STH; is this a question which needs to be addressed given the emphasis on the need to treat adults as well as children if the impact of STH treatments are to be maximised*[40–42]*?*
- ➢*What impact does STH deworming using albendazole and mebendazole of school age children have on LF either pre or post MDA interventions*[41, 42]*?*

### *Loa loa*- the monster in the room

During the early phase of the CDTi programme in Cameroon, severe adverse events (SAEs) were reported [43] from areas co-endemic with the filarial parasite *Loa loa*. Some individuals who had high densities of *L. loa* microfilaria (≥30,000/ml) and who received ivermectin developed encephalopathic reactions following treatment [44]. Following the reports of SAEs in Cameroon, safety concerns prevented the wider expansion of CDTi programmes to eliminate onchocerciasis and LF in areas co-endemic with *L. loa* where there is the possibility of treating over 90% of the population who are not infected*.* The situation remains a major challenge and has prevented the initiation of LF programmes [3, 7] using ivermectin with albendazole in MDA. Treatment with ivermectin is continuing in areas of meso and hyperendemic onchocerciasis where it is considered the risks of an SAE is justified due to the long-term effects of onchocerciasis in the population. In these areas, strict guidelines were put in place for the early identification and management of SAE cases, reducing the mortality and morbidity considerably.

The problems presented by the risk of SAEs have required the search for alternative elimination strategies for deployment in LF/*L. loa* co-endemic areas. This has resulted in the recommendation that twice yearly albendazole be implemented together with the expanded use of bed nets/LLINs, which are now widely used for malaria control [3, 45]. To address the problems of SAEs it was necessary to identify areas of highest risk of loiasis in onchocerciasis endemic areas; a field-applicable non-invasive rapid assessment technique was developed (RAPLOA) to address this. A survey was administered on the ability of villagers to identify the presence of the *L. loa* adult in the eye. A questionnaire which asked about the history of eye worm using a restricted definition was developed, and results were based on the proportion of individuals who had a history of eye worms confirmed by a photograph or a Calabar swelling that lasted 1–7 days [46, 47]. The studies indicated that the rapid assessment method produced a strong correlation between parasitological indices and those obtained by questionnaire.

Recent studies on SAEs in Bas Congo, DRC [48], have shown that the events are associated with areas considered to be at lower risk with a RAPLOA prevalence of between 20-40%, but were found to be 10–16 times higher than elsewhere. This implies that the RAPLOA estimated prevalence is not necessarily the primary indicator of SAE risk, but rather the intensity of infection in individuals when they have high parasitaemias of *L. loa* (≥30,000 mf/ml) [49]. This is a feature not predicated in any overall prevalence data [50] - but something that needs to be examined on a finer spatial scale taking into account human population densities, age-sex compositions, movement and migration, and local environmental factors such as vegetation and forest cover. These are important to *Chrysops spp.* vectors and their potential to breed, bite, transmit and maintain infection in the community [51].

**Figure 2:**
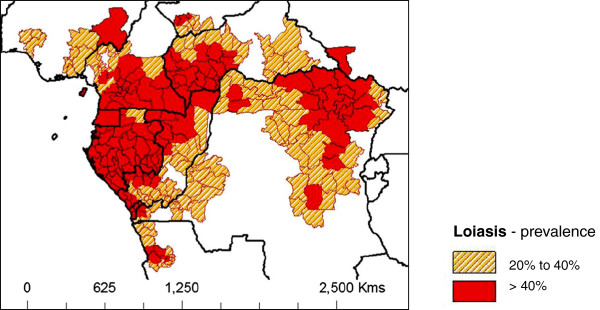
**Loiasis intermediate and high risk subnational areas.** Risk coding based on loiasis map by Zouré *et al.*[47] and created using country sub-national boundaries in ArcGIS (ESRI, Redlands, CA).

Hence, given the wide geographical range of the RAPLOA 20-40% intermediate prevalence zones across Central and West Africa (Figure 2), the risk of *Loa* encephalopathies are likely to be more extensive than previously estimated. This is important in the context of treatment in onchocerciasis hypoendemic/low transmission areas and where onchocerciasis elimination requires MDA over perhaps at least a 10 year period and where LF prevalence is also above 1% and where MDA should be initiated. Thus, the recommended strategy of twice yearly albendazole in combination with bed nets/LLINs for LF programmes [45] will extend through areas of *L. loa* endemicity irrespective of RAPLOA risk mapping as the sensitivity of the 20-40% intermediate zone is based purely on a questionnaire [46, 52]. Within all these co-endemic zones the impact on LF transmission over recent years needs to be better estimated based on recent bed nets/LLIN coverage, usage and efficacy [29, 41]. This is a major area currently lacking attention within many LF programmes. Similarly, there is little information of the extent and impact of CDTi activities to date where there may be or have been *W. bancrofti* endemicity.

**Figure 3:**
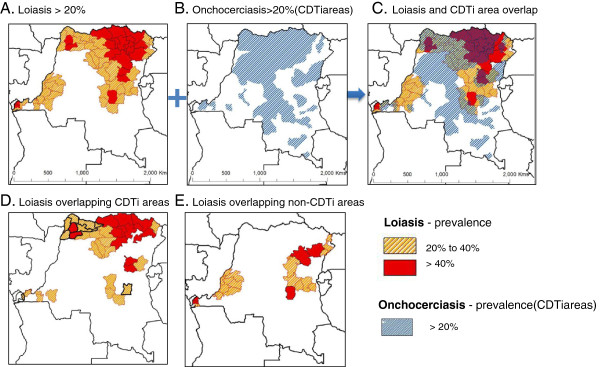
**Maps of loiasis intermediate and high risk areas geographically overlapping with CDTi and non-CDTi areas.** Orange shading indicates loiasis intermediate prevalence areas of 20-40%. Red shading indicates loiasis high prevalence areas of > 40%. Blue shading indicates onchocerciasis > 20% and areas targeted with ivermectin, also known as CDTi areas. **A**. Loiasis >20% **B**. Onchocerciasis >20% (CDTi areas) **C**. Loiasis and CDTi area overlap **D**. Loiasis overlapping CDTi areas **E**. Loiasis overlapping non-CDTi areas.

Here we use DRC as a case study to highlight the complexities and challenges for the new LF programme scaling up MDA across large hard-to reach- *L. loa* co-endemic areas [47]. Figure 3 shows the wide loiasis and CDTi overlapping distributions across the country (Figure 3A-C), and highlights that many intermediate/high risk loiasis areas have already received multiple rounds of ivermectin (Figure 3D), and that there are far fewer areas than expected that are totally drug naïve and non-CDTi areas (Figure 3E). However, what is important to note is that in the drug naïve areas in the east of the country, there is a significant lack of bed nets with a range of geographical and infrastructure barriers to reaching this vulnerable population [53]. We summarise below the key questions we consider must be addressed over the coming years by LF programmes in LF/onchocerciasis/*L. loa* co-endemic areas.

The key programmatic and research questions which can be posed to address the multidimensional complexity of the current problems of MDA in LF/ onchocerciasis/ *L. loa* co-endemic areas

**Compliance/adherence, coverage and cooperation**

- ➢*In LF/L. loa co-endemic areas that overlap CDTi areas, which have already received multiple rounds of ivermectin, what does adding albendazole do for compliance /adherence?*
- ➢*Have coverage levels of ivermectin through the CDTi platform been high enough to avert SAEs with the rigor of a new LF programme, which may reach populations previously untreated or sub-optimally treated?*
- ➢*How does the LF programme scale up the alternative strategy of twice yearly albendazole and bed nets/LLINs in LF/L. loa co-endemic /non-CDTi areas, especially in remote inaccessible areas with limited bed nets /LLIN coverage and where no MDA has yet been implemented*[53]*?*
- ➢*How does the LF programme engage with the malaria control programme to coordinate, distribute and maintain adequate coverage of LLINs where they are needed most; who will monitor insecticide resistance?*[54]*; do potential SAE areas overlap malaria endemic areas?*

**Consequential impact of alternative strategies**

- ➢*What will be the impact of the alternative strategy including twice yearly albendazole for L. loa endemic areas on STH distribution? How does the LF programme engage with new STH programmes scaling up in country, share resources and measure impact across the multiple diseases?*
- ➢*Will the alternative strategy of twice yearly albendazole for L. loa have the same impact across a diverse range of ecological and epidemiological settings; how will it be measured and monitored?*
- ➢*What will be the impact of the alternative strategy, including twice yearly albendazole be on O. volvulus, especially low transmission/hypo-endemic areas, in the absence of ivermectin?*

## Review and conclusions

The NTD community must recognise that implementation of the current strategy through a single disease specific focus, whilst expanding through integrated implementation strategies, must adapt its approach to the multidimensional challenges now presented. This is indicated by the wider implications of the impact of the drugs already distributed, the long term impact of ivermectin and albendazole on STH where some 200 million treatments are given annually in Africa for onchocerciasis and LF [7] and the impact of bed nets/LLINs on *W. bancrofti* and its transmission [13–16, 29]. For STH, whilst the current focus is on the treatment of school age children, the need to treat pre-school aged children and those pupils not attending school needs significant consideration [55]-for example are they receiving treatment through onchocerciasis and LF programmes if under 90 cms height. These important cohorts of the population will require treatment and systematic surveillance.

Recently, the importance of treating adults and the need for water and sanitation (WASH) programmes has been emphasised if “elimination” is to be achieved [56], but the impact of onchocerciasis and LF interventions which have been distributed for almost two decades for onchocerciasis and a decade for LF with over 2 billion treatments given cumulatively in Africa must be factored into the evaluation, and mapping models to reflect impact on one hand, and accuracy and sensitivity of any mapping to determine future interventions, on the other.

We believe the questions we pose are operationally critically important in the context of the established Roadmap targets [28]; these issues affect strategy, policy, operational and implementation issues and are particularly critical in the context of mapping, planning, monitoring and evaluation, surveillance and elimination.
